# Remarkable Phytochemical Characteristics of Chi-Nan Agarwood Induced from New-Found Chi-Nan Germplasm of *Aquilaria sinensis* Compared with Ordinary Agarwood

**DOI:** 10.1155/2021/5593730

**Published:** 2021-04-10

**Authors:** Meng Yu, Yangyang Liu, Jian Feng, Deli Chen, Yun Yang, Peiwei Liu, Zhangxin Yu, Jianhe Wei

**Affiliations:** ^1^Key Laboratory of Bioactive Substances and Resources Utilization of Chinese Herbal Medicine, Ministry of Education & National Engineering Laboratory for Breeding of Endangered Medicinal Materials, Institute of Medicinal Plant Development, Chinese Academy of Medical Sciences and Peking Union Medical College, Beijing 100193, China; ^2^Hainan Provincial Key Laboratory of Resources Conservation and Development of Southern Medicine & Key Laboratory of State Administration of Traditional Chinese Medicine for Agarwood Sustainable Utilization, Hainan Branch of the Institute of Medicinal Plant Development, Chinese Academy of Medical Sciences and Peking Union Medical College, Haikou 570311, China

## Abstract

Wild Chi-Nan agarwood is regarded as the highest quality agarwood from *Aquilaria* spp. However, the comprehensive research on chemical composition of wild Chi-Nan agarwood is limited. An integrated strategy using SHS-GC-MS and UPLC-Q/Tof-MS was applied to explore the phytochemical characteristics of a kind of agarwood induced from a newly identified germplasm of Chi-Nan *A. sinensis.* Progenesis QI and MS-Dial were used to preprocess the UPLC-Q/Tof-MS and GC-MS raw data, respectively. Principle component analysis (PCA) and orthogonal partial least squares to latent structure-discriminant analysis (OPLS-DA) models were built to discriminate Chi-Nan agarwood from ordinary agarwood and to screen potential distinguishing components between them. In this study, we clarified the distinguishing differences between Chi-Nan agarwood and ordinary agarwood. The difference is mainly manifested in the average contents of 2-(2-phenylethyl)chromone and 2-[2-(4′-methoxybenzene)ethyl]chromone, which are 170 and 420 times higher in Chi-Nan agarwood than in ordinary agarwood, respectively, while the contents of 5,6,7,8-diepoxy-2-(2-phenylethyl)chromones(DEPECs), 5,6-epoxy-2-(2-phenylethyl)chromones(EPECs), and 5,6,7,8-tetrahydro-2-(2-phenylethyl)chromones(THPECs) such as agarotetrol are extremely low. The content of the main sesquiterpenes in Chi-Nan agarwood was higher than that in ordinary agarwood, especially in regard to guaiane and eudesmane derivatives. In addition, there were significant differences in the contents of low-molecular-weight aromatic compounds such as 2-methyl-4H-1-benzopyran-4-one, 4-methoxybenzaldehyde, and 2-hydroxybenzaldehyde between Chi-Nan agarwood and ordinary agarwood. All the mentioned main chemical characteristics of this new Chi-Nan agarwood were coincident with those of the rare wild Chi-Nan agarwood from *A. malaccensis*, *A. sinensis*, and *A. crassna*. We reported differences in 2-(2-phenylethyl)chromones, sesquiterpenes, and low-molecular-weight aromatic compounds between Chi-Nan agarwood and ordinary agarwood from *A. sinensis* for the first time; it is necessary to evaluate the agarwood from the new-found Chi-Nan germplasm.

## 1. Introduction

Agarwood is resinous wood obtained from wounded *Aquilaria* tree, which is a genus belonging taxonomically to the Thymelaeaceae family [1]. The main constituents in agarwood are volatile constituents and semi-volatile components [2]. The former include low-molecular-weight aromatic compounds and sesquiterpene derivatives, and the latter principally consist of 2-(2-phenylethyl)chromone derivatives [3]. Sesquiterpenes, including agarofurans, cadinanes, eudesmanes, eremophilanes, guaianes, and agarospiranes, are considered to be the prominent contributors to agarwood aroma [4]. 2-(2-phenylethyl)chromone monomers can be divided into four categories based on the A ring of 2-(2-phenylethyl)chromones, namely, 5,6,7,8-tetrahydro-2-(2-phenylethyl)chromones (THPECs), diepoxy-tetrahydro-2-(2-phenylethyl)chromones (DEPECs), epoxy-tetrahydro-2-(2-phenylethyl)chromones (EPECs), and flindersia type 2-(2-phenethyl)chromones (FTPECs) [2, 5].

Chi-Nan agarwood (CNA), a high-quality agarwood, is also called Qi-Nan or Jar-Nan in China, *Kanankoh* or *Kyara* in Japan, and *Tagara* in India [6]. In China, wild CNA is considered representative of high-quality agarwood, whose price is increasing up to thousands of RMB yuan per gram [7, 8]. Wild Chi-Nan agarwood is valued for its mysterious and elegant oriental odour that could be obviously smelt without heating, which make it discriminate from other kinds of agarwood. Investigations on wild CNA have rarely been reported, in contrast to abundant reports related to ordinary agarwood. In 1985, Hashimoto proved that benzaldehyde and 4-methoxybenzaldehyde originated from the pyrolysis of 2-(2-phenylethyl)chromone and 2-[2-(4′-methoxyphenyl)ethyl]chromone, respectively, in the neutral part of *Kyara* [9]. Ishihara discovered that the relative contents of 2-(2-phenylethyl)chromone and 2-[2-(4′-methoxyphenyl)ethyl]chromone from *Kanankoh* (*A.agallocha,* synonym: *A. malaccensis*) [10] smoke were much higher than those from Jinkoh [11]. In addition, taking four types of wild CNA (*A. sinensis* and *A. agallocha*) as materials, Dai showed that there were abundant 2-(2-phenylethyl)chromones and 2-[2-(4′-methoxyphenyl)ethyl]chromones [6, 12]. It is not difficult to notice that previous studies took only the 2-(2-phenylethyl)chromones into account. In addition, Chinese pharmacologist Xie Zongwan described the morphological characteristics of CNA but failed to provide information on the original plant [13]. Due to the confusion regarding the original plant, relevant information on wild CNA has seldom been described in the mentioned studies.

In recent years, a kind of special agarwood germplasm of *A. sinensis* has been introduced and domesticated from the wild population and propagated by grafting with ordinary germplasm of *A. sinensis*. Agarwood with more than 40% of the alcohol soluble extractive content can be obtained after this kind of tree is wounded with the drilling method over one year. However, for ordinary germplasm of *A. sinensis*, it is almost impossible to obtain agarwood with more than 10% of the alcohol soluble extractive content with the same inducing method [14]. Because of the similarity of characteristics such as appearance, form, texture, color, smell, and taste to wild CNA, this new-found agarwood is also called CNA. To date, there has been only one report by Li [15]in which the chemical properties of this kind of CNA have been tested by TG-FTIR and HS-GC-MS, and these authors named it *Kynam*. However, the authors did not clarify the specific components to distinguish CNA in terms of fragrance and the chemical characteristics of CNA. Here, gas chromatography-mass spectrometry (GC-MS) and liquid chromatography-mass spectrometry (LC-MS) were used to analyze the differences between CNA induced from new-found germplasm and ordinary agarwood (OA) to obtain a deep knowledge of phytochemical characteristics.

## 2. Materials and Methods

### 2.1. Agarwood Materials

Thirteen agarwood samples were analyzed as shown in Table 1 and Figure S1. They are divided into CNA (Chi-Nan agarwood) and OA (ordinary agarwood) group. The seven CNA samples were collected from new-introduced germplasms *A. sinensis* tree that were provided by three farmers from different planting bases of Hainan and Guangdong province in China. As for the OA group, we chose six ordinary agarwood induced with three kinds of common agarwood-inducing methods, including whole-tree agarwood-inducing technique (Agar-Wit) [16], burning-chisel-drilling (BCD), and wild.

### 2.2. Chemical Reagent

Al_2_O_3_ powder as dispersant (High purity, Tianjin Guangfu Fine Chemical Research Institute, China) and retention index marker alkanes (AccuStandard, USA, C9–C40) were used in GC-MS, as well as anhydrous alcohol (AR, Xilong Science Co., Ltd, China), and acetonitrile (Merck HPLC grade, Darmstadt, Germany).

### 2.3. Sample Preparation

For all the samples described in Section 1, agarwood material sections, chips, or blocks were ground into powder with liquid nitrogen. Agarwood powder (0.2 g) was extracted with 10 mL of 50% ethanol by means of sonication at room temperature for 30 min. Then, the supernatant was used as the sample reserve solution. Through preexperiments, we found that the content of 2-(2-phenylethyl)chromones and 2-[2-(4′-methoxyphenyl)ethyl]chromones in CNAwas too high to analyze, so we diluted the test solution 10 times before analysis.

Agarwood powder was put into a 20 ml headspace bottle, 0.2 g Al2O3 was added as the dispersant [17], and the solution was analyzed in the headspace sampler according to the following conditions: headspace heating temperature: 180°C; transmission line temperature: 185°C; injection probe temperature: 190°C; headspace heating time: 40 min; and N_2_ purging time: 30 s. For each of the samples, 1 ml of headspace gas was used for GC-MS analysis. The retention index marker alkanes (C9–C40) were analyzed by the same program in GC-MS.

### 2.4. LC-Q/Tof-MS Analysis Condition

LC-MS was performed using an ultrahigh-performance liquid chromatography (UPLC) system (H-class, Waters, USA) coupled with a quadrupole time-of-flight tandem spectrometer (Xevo G2-XS, Waters, USA). Separation was performed using a Waters Acquity UPLC BEH C18 column (3.0 mm × 100 mm, 1.7 *μ*m, Waters, USA). The column temperature was 30°C. The detection wavelength was set at 196 nm for all the tested compounds, and the mobile phases were acetonitrile (*A*) and water (*B*). A gradient elution was used: 0 min, 20% (*v*/*v*) *A*; 20 min, 50% (*v*/*v*) *A*; 30 min, 20% (*v*/*v*) *A*. The mobile phase was established at a flow rate of 0.2 mL·min^−1^, and the injection volume was 5 *μ*L.

The nebulization gas was set to 600 L·h^−1^ at a temperature of 350°C, and the cone gas was set to 50 L·h^−1^. The source temperature was set to 110°C. The capillary voltage and cone voltage were set to 3500 V and 30 V, respectively. The data acquisition rate was set to 0.3 s with a 0.1 s interscan delay. Data between *m/z* 50 and 1200 were recorded in positive ion mode. The quality axis was corrected by sodium formate, and the quality of leucine enkephalin was corrected in real time.

### 2.5. SHS-GC-MS Analysis Conditions

GC-MS analysis was performed with a TSQ GC system (Agilent Technologies, USA). The chromatographic separation was conducted with a DB-5MS capillary column (30 m × 0.25 mm i.d., 0.25 mm film thickness, Agilent Technologies, USA). The GC/MS interface temperature was maintained at 250°C. Ultrahigh purity helium (99.9995%) was used as the carrier gas at a flow rate of 1 mL·min^−1^. The column temperature program was set as follows: 1 min at the initial temperature of 50°C, which was subsequently ramped to 143°C at a rate of 15°C·min^−1^; 10 min at 143°C, which was ramped to 155°C at a rate of 1°C·min^−1^; 0 min at 155°C, which was ramped to 225°C at a rate of 25°C·min^−1^; 7 min at 225°C, which was ramped to 300°C at a rate of 25°C·min^−1^; then the temperature was held at 300°C for 5 min. The ratio of gas flowing out of the chromatographic system and gas flowing into the chromatographic column after sample gasification was 5 : 1.

### 2.6. Data Preprocessing and Statistical Analysis

UPLC-Q/Tof-MS raw data were imported into Progenesis QI (Waters, USA) for preprocessing, while GC-MS raw data were converted and preprocessed by MS-Dial (NSF-JST, Japan). Statistical analysis was carried out by SIMCA-P (version 14.1, Umetrics, Umea, Sweden) on the basis of data matrixes. Unsupervised principal component analysis (PCA) was used to identify similarities or latent differences between groups. Data plotted using PC revealed intersample relationships via their spatial proximity. Orthogonal projection to latent structure discriminant analysis (OPLS-DA) was then implemented to detect maximum information from the data set and to distinguish the components induced by different groups. Distinguished compounds were screened by analyzing the Variable Importance in the Projection (VIP) and S-plot. Finally, Student's *t*-test (*p* < 0.05) was used to screen the significant variables with SPSS R26 (IBM, USA).

## 3. Results

### 3.1. Phytochemical Characteristics of CNA Based on LC-MS

**Figure 1:**
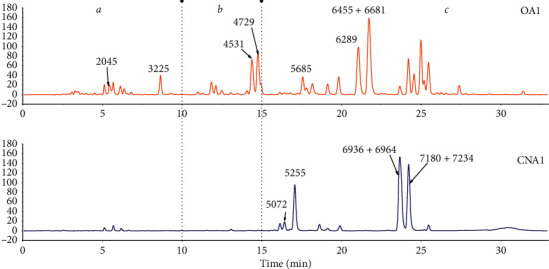
The total ion chromatograms of Chi-Nan agarwood (CNA1) and ordinary agarwood (OA1) acquired by LC-MS. (*a* zone: 0–10 min; *b* zone: 10–15 min; *c* zone: 15–30 min).

Based on the high-resolution mass spectrometry and fragment ion information obtained in the experiment, thirty 2-(2-phenethyl)-chromones in thirteen batches of samples were identified according to the methods used in previous studies [18–20], as shown in Table S1. They were distributed into three parts of the total ion chromatograms in Figure 1. It is noteworthy that, between 0 and 10 min (zone *a*), the main components detected were THPECs, while EPECs/DEPECs flowed out from 10–15 min (zone b). The FTPECs and dimers flowed out after 15 min of the elution gradient (zone c). Historically, the composition of CNA is relatively simple, especially before 15 min, which indicates that FTPECs are the predominant type of 2-(2-phenethyl)chromones in CNA.

**Figure 2:**
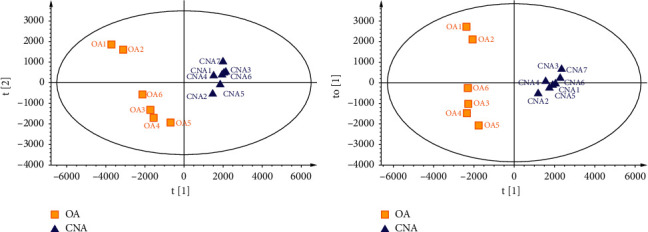
PCA (a) and OPLS-DA (b) scores plots for the first two components of Chi-Nan agarwood (filled triangle)and ordinary agarwood (filled square) data analyzed by LC-Q/Tof-MS.

Thus, we carried out PCA to obtain an objective understanding of LC-MS data, and the results are shown in Figure 2(a). The two PCs described 72.9% variance, of which the first PC (PC1) accounted for 57.3% and PC2 accounted for 15.6% of the total variance. The CNA mainly gathered on the positive side of PC1, and the OA was distributed on the negative side of PC1 which proved that the two groups had obvious differences in the composition of 2-(2-phenethyl)chromones. Then, supervised OPLS-DA was subsequently used. As depicted in Figure 2(b), the CNA samples are clearly separated from the OA samples [R2X (cum) = 0.727; R2Y (cum) = 0.977; Q2 (cum) = 0.952]. One orthogonal component was calculated. The validation plot obtained from 200 permutation tests confirmed the validity of this OPLS-DA model. The criteria for validity include the following: all the permuted R2 and Q2 values to the left are lower than the original points to the right, and the regression line of the Q2 points intersects the vertical axis (on the left) at or below zero.

### 3.2. Phytochemical Characteristics of CNA Based on GC-MS

**Figure 3:**
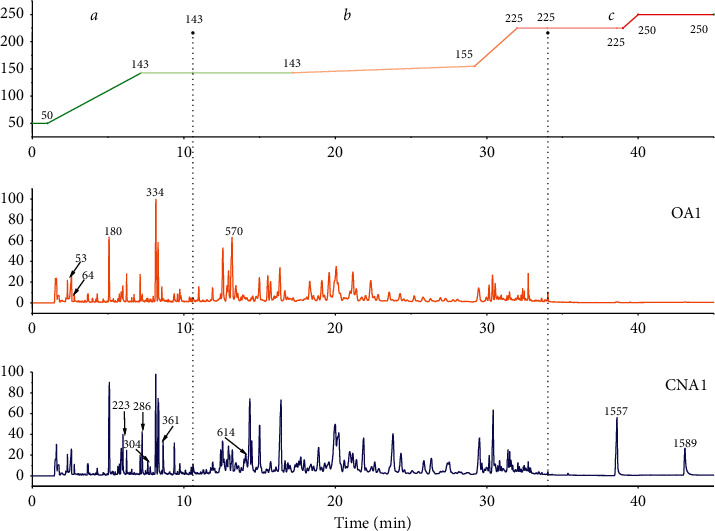
The total ion chromatograms of typical agarwood samples (OA1 and CNA1) acquired by SHS-GC-MS and the corresponding temperature gradientvariety diagram (*a* zone: 0–10.6 min; *b* zone: 10.6–34.0 min; *c* zone: 34.0–45 min).

To obtain a chemical profile of the violate constituents of agarwood, an analytical method based on SHS-GC-MS was developed. The TIC chromatographs of representative samples (OA1 and CNA1) and the plot corresponding to the temperature program are shown in Figure 3. With the application of Agilent MassHunter Quantitative Analysis 10.0, 101 compounds were identified by searching NIST14 in the MS data and retention index. Tentative identification with similarity over 80% and retention index within ±20 is listed in Table S2. Figure 3 shows that low-molecular-weight aromatic compounds, sesquiterpenes, and chromones flow out in order during different temperatures. Among them, mainly low-molecular-weight aromatic compounds were detected when the temperature was above 50°C (zone *a*). When the temperature ranges from 142°C to 225°C (zone *b*), sesquiterpenes and their thermal cracking fragments flow out of the column. Finally, low-polarity 2-(2-phenethyl)-chromones can be detected when the temperature rises up to 225°C until 38.6 min (zone *c*).

**Figure 4:**
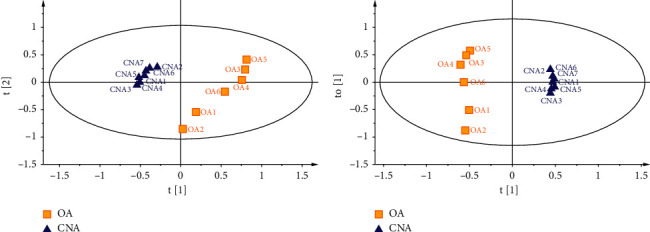
PCA (a) and OPLS-DA (b) scores plot for the first two components of Chi-Nan agarwood (filled triangle) and ordinary agarwood (filled square) data analyzed by GC-MS.

Mutual projections of factor scores for the first two PCs are presented in Figure 4(a), and these scores described 35.8% (PC1) and 16.7% (PC2) of the variability in the data when ellipse hoteling was set as 95% (70.1% can be explained by the four components). Thirteen batches of agarwood samples could be readily divided into two different groups, which indicated that the content and distribution of components were different between CNA and OA. The CNA samples were clustered closer to each other than to the OA samples on the negative side of PC1, which indicates that the differences between the groups are relatively minor.

To identify distinguishing compounds, the data were further processed with orthogonal partial least squared discriminant analysis (OPLS-DA). The classification results are shown in Figure 4(b). The CNA group could be clearly separated from the OA group. The values of R2X (cum) and R2Y (cum) are 0.628 and 1, respectively, which is an important parameter to show how much data contributed to creating the model. In addition, Q2 (cum) was 0.956, indicating that the OPLS-DA model had high predictability. To guard against model overfitting, permutation tests with 200 iterations were carried out.

**Figure 5:**
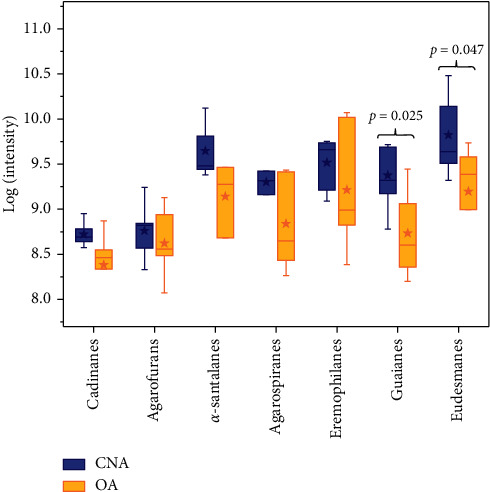
Comparison of sesquiterpenes in different configurations between the CNA (colored blue) and OA (colored yellow) (the stars on the boxes represent mean values of different types of sesquiterpenes). The stars on the boxes represent mean values. The median is drawn as a black horizontal line inside the box.

Furthermore, we analyzed the distribution of different types of sesquiterpenes in the samples. Finally, a total of seventeen types of sesquiterpenes were identified in thirteen batches of samples. Among them, seven predominant types of sesquiterpenes, including *α*-santalanes, agarospiranes, guaianes, eremophilanes, eudesmanes, cadinanes, and agarofurans, were used for successive analysis. Following the logarithmic conversion of the total peak area of various sesquiterpenes, Figure 5 reflects the distribution of different configurations of sesquiterpenes between CNA and OA. The stars on the boxes represent mean values, and the results indicate that CNA possesses a higher concentration of all sesquiterpenes than OA. The length of the box, which indicates the interquartile range, clearly shows that CNA possesses greater dispersion. Obviously, eudesmane derivatives are most abundant in CNA, while eremophilane derivatives comprise the majority of sesquiterpenes in OA. Comparatively, the concentrations of guaianes and eudesmanes were significantly different (*p* < 0.05) between the two groups.

### 3.3. The Distinguishing Components between CNA and OA

**Figure 6:**
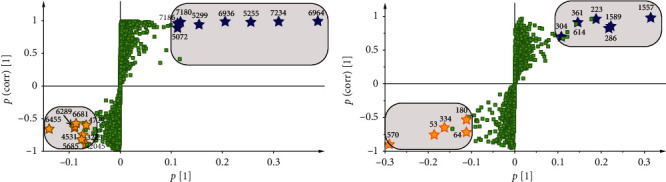
S-plot at the first component used in potential distinguished components selection based on LC-Q/Tof-MS (a) and GC-MS (b), constituent ions with VIP value >4 were marked with a black square, compounds marked with yellow stars are richer in CNA, and dark blue marked compounds are richer in OA.

To determine the differences in the composition of 2-(2-phenethyl)-chromones, 13 potential distinguishing components were identified, as shown in Table 2. The S-plot derived from the OPLS-DA model comparing the two groups is shown in Figure 6(a). The VIP value reflects the influence of every compound's ion on the classification. Variables with a VIP value >1 have an above-average influence on the explanation of the *Y* matrix. In our research, we adjusted VIP up to 5 to evaluate significant variables according to the practical situation. The qualified variables that meet the above criteria are the best possible distinguishing components labeled by a gray square. Subsequently, these components were used for Student's *t*-test.

Table 2 shows seven identified significant components, including two DEPECs, two THPECs, and three FTPECs with higher contents in OA; these components are highlighted with blue stars. The *p* values of compounds **2045**, **3225**, and **5685** were less than 0.01, which means that the differences in these constituents were significant. Likewise, all 6 identified significant components were FTPECs and dimeric PECs in CNA. Moreover, all the constituents' *p* values were below 0.001. It should be noted that the average peak areas of 2-(2-phenylethyl)chromone and 2-[2-(4′-methoxybenzene)ethyl]chromone are 170 and 420 times higher in CNA than in OA, respectively, as shown in Table S2.

We applied the same method to select the distinguishing volatile constituents from agarwood. According to the specific conditions of this experimental variable, the VIP threshold was adjusted to 4. Based on the above conditions, the compounds in the rectangular frame, as shown in Figure 6(b), are highlighted. Student's *t*-test was performed in succession, and variables without significant differences between the two groups (*p* > 0.05) were eliminated. The remaining compounds (marked with stars in Figure 6(b)) were selected for identification. Detailed information on the twelve distinguishing components is listed in Table 3. The most noteworthy differences between CNA and OA are in low-weight aromatic compounds and 2-(2-phenylethyl)chromones. Regarding low-weight aromatic compounds, compounds **53**, **64**, **180**, **334**, and **570** were abundant in OA, while compounds **223**, **286**, **304**, **361**, and **614** were abundant in CNA. Remarkably, the *p* values of 2-(2-phenethyl)-chromone and 2-[2-(4′-methoxyphenyl)ethyl]-chromone were below 0.001, which indicates that the contents of these two compounds are significantly different between CNA and OA.

## 4. Discussion

### 4.1. The Differences in 2-(2-Phenylethyl)chromones between CNA and OA

As the results demonstrate, the 2-(2-phenylethyl)chromones in CNA induced from new-found Chi-Nan germplasm are relatively simple and all belong to FTPECs. Noticeably, the contents of 2-(2-phenylethyl)chromone and 2-[2-(4′-methoxybenzene)ethyl]-chromone are hundreds of times higher in CNA than in OA. Additionally, Japanese researchers discovered that the relative content of two constituents accounted for 5.83% and 1.59% in *Kanankoh* (wild CNA), while they accounted for only 0.28% and below 0.05% in *Jinkoh* (OA) [11]. Dai reported that the relative contents of 2-(2-phenylethyl)chromone and 2-[2-(4′-methoxyphenyl)ethyl]-chromone were extremely high in the ether extract of four kinds of wild CNA. Therefore, we considered 2-(2-phenylethyl)-chromone and 2-[2-(4′-methoxyphenyl)ethyl]-chromone as phytochemical characteristic constituents to distinguish CNA. Such characteristics can also account for the high alcohol soluble extractive content, as described in previous studies [15].

However, some 2-(2-phenylethyl)chromones, such as DEPECs, EPECs, and THPECs, exist in agarwood apart from FTPECs, especially THPECs, which are specific compounds that have so far been detected only in agarwood [21]. We found that the amount and content of THPECs and DEPECs in OA, such as compounds **2045**, **4531**, **3225**, and **4729**, especially agarotetrol (**2045**), were higher. Agarotetrol has been designated as an index component in the quality control of content determination in medicinal agarwood according to the *Chinese Pharmacopoeia* 2020. In contrast, the content of THPECs in CNA was extremely low, especially in agarotetrol. Thus, CNA fails to reach the requirement of the *Chinese Pharmacopoeia* 2020 on the basis of the content of agarotetrol.

### 4.2. Distinguishing Differences in Sesquiterpenes and Low-Weight Aromatic Compounds between CNA and OA

Agarwood is processed into perfumes and incenses in addition to being used as a raw material in traditional and modern medicines. Sesquiterpene volatile constituents and low-weight aromatic compounds are the major volatile constituents in agarwood [2].Therefore, SHS was used as the pretreatment method to analyze the volatile components of agarwood to replicate the fumigation. In our results, 69 sesquiterpenes were detected and identified from CNA and 43 sesquiterpenes from OA. However, it is noteworthy that the contents of guaianes and eudesmanes derivatives in CNA were superior to those in OA. It has been reported that guaiane and eudesmane derivatives accounted for the majority of sesquiterpenes in wild CNA according to investigations of Ishihara et al. [22] and Yang et al. [23]. Even more importantly, many of them were reported to possess a pleasant fragrance [2, 12]. Guaianes and eudesmanes seem to contribute to the gorgeous and elegant character of CNA by contributing to their odoriferous properties [18]. Consequently, guaianes and eudesmanes may be significant contributors to the unique fragrance of CNA. Of course, we need to carry out relevant studies to specify the characteristics in terms of sesquiterpenes so that we can explain the fragrance of the CNA more perspicuously.

It is meaningful that we found that several low-weight aromatic compounds are similar to the partial structure of FTPECs, such as 4-methoxybenzene-methanol (**361**), 2-hydroxybenzaldehyde (**223**), and 1-(2-hydroxyphenyl)ethenone (2**86**). Moreover, the contents of these compounds were higher than those in OA, so it is reasonable for us to speculate that the above low-weight aromatic compounds partly originate from the pyrolysis of 2-(2-phenylethyl)-chromone and 2-[2-(4′-methoxybenzene)ethyl]-chromone. Similarly, the high content of 4-methoxybenzaldehyde (**304**) in CNA has the same explanation. In fact, Hashimoto found that2-(2-phenylethyl)chromone and 2-[2-(4′-methoxyphenyl)ethyl]chromone could pyrolyze at 150°C for 6 h to produce benzaldehyde and 4-methoxybenzaldehyde, respectively [9]. It may be difficult to understand the origin of abundant benzaldehyde in OA. However, Takamatsu proved that agarotetrol could produce pyrolysis products such as 4-phenyl-2-butanone and benzaldehyde with HS-SPME-GC-MS analysis when heated to 190°C–200°C [19]. Ishihara et al. found that smoke from agarwood contained a small amount of pulp wood pyrolysis products such as acetic acid, benzaldehyde, and vanillin when *Kanankoh* (CNA in Japanese) was heated at 180–210°C by an alcohol lamp [11]. Therefore, we can hypothesize that the non-resinous part and agarotetrol in OA results in high concentrations of benzaldehyde (**180**) and acetic acid (**53**). In addition, we discovered that the content of 4-phenyl-2-butanone (**334**) in OA is nearly 1.5 times as high as that in CNA by calculating the peak area shown in Table S2. This is probably related to the biosynthesis of 2-(2-phenylethyl)chromones according to Liao's research [20]. In conclusion, there were considerable discrepancies in low-weight aromatic compounds between CNA and OA.

### 4.3. The Phytochemical Characteristics of New-Found CNA Are Consistent with Those of Wild CNA

Considering the chemical composition and application characteristics of agarwood, we comprehensively explored the phytochemical characteristics of CNA induced from new-found Chi-Nan germplasm of *A. sinensis*. Comparatively, CNA and OA showed dramatic differences in chemical composition. Notably, the phytochemical characteristics of CNA were consistent with those of wild CNA reported by Chinese and Japanese researchers in terms of phytochemical characteristics. In addition, we analyzed a wild CNA sample (CNA8) under the same conditions, and the corresponding results are shown in Fig.  S2-S3. Therefore, it is acceptable for us to speculate that CNA in this study is a kind of agarwood that is similar to wild CNA originating from *A. sinensis* or *A. malaccensis*, which is reported by both foreign and domestic researchers [11, 12]. Thus, this is the first study to comprehensively reveal the distinguishing phytochemical characteristics of volatile and semi-volatile components of this newly identified CNA, and the findings indicated that CNA may originate from Chi-Nan germplasm of *Aquilaria* spp. rather than a unique environment, induction method, or species. We also identified the original plant of this CNA via DNA barcoding technology as *A. sinensis*. The relevant conclusions will be published in another paper.

## 5. Conclusion

We demonstrated the historical phytochemical characteristics of CNA for the first time. Here, we reveal distinguishing differences in terms of 2-(2-phenylethyl)chromones, sesquiterpenes, and low-molecular-weight aromatic compounds between CNA and OA. The main chemical characteristic of CNA is the single composition of 2-(2-phenylethyl)chromones, which is characterized by extremely high contents of 2-(2-phenylethyl)chromone and 2-[2-(4′-methoxybenzene)ethyl]chromone in CNA. Regarding the different types of 2-(2-phenylethyl)chromones, CNA mainly contains FTPECs, while the contents of DEPECs, EPECs, and THPECs, such as agarotetrol, are extremely low. The content of low-molecular-weight aromatic compounds in the smoke of agarwood detected by heating from the pyrolysis of FTPECs was higher than that in OA, especially for 4-phenyl-2-butanone, benzaldehyde, 4-methoxybenzaldehyde, and 2-hydroxybenzaldehyde. Nevertheless, the concentrations of acetic acid, benzaldehyde, and 4-phenyl-2-butanone were much higher in OA than in CNA. Remarkably, the guaiane and eudesmane derivatives with higher content in CNA were likely to be the crucial contributors to the unique fragrance of CNA when used as incense.

Due to the significant differences between CNA and OA, it can be inferred that CNA may originate from Chi-Nan germplasm of *Aquilaria* spp., which can produce CNA in a short period of time without a special environment, place of origin, or method. The unique phytochemical characteristics of CNA may be related to the genetic information of the original plant germplasm or endophytic fungi. However, the biosynthesis mechanism of the abovementioned chemical characteristics needs further research for clarification.

## Figures and Tables

**Table 1:** Sample information.

Num.	Species	Agarwood induction method	Place of production	Description	Sinkage
CNA1	*A. sinensis*	3-year-old Chi-Nan germplasm by drilling for 15 months	Ding'an, Hainan province	Irregular pieces, black brown resin bands alternate with yellow white wood stripes, sufficient resin, rich of aromas, acrid in taste, soft and glutinous	x
CNA2	*A. sinensis*	3-year-old Chi-Nan germplasm by drilling for 14 months	Ding'an, Hainan province	Irregular strips, brown resin bands and white wood are distributed alternately, strong fragrance, cool feeling, bitter in taste, hard texture, slightly sticky	x
CNA3	*A. sinensis*	5-year-old Chi-Nan germplasm by drilling for 18 months	Ding'an, Hainan province	Irregular pieces, black brown resin scatter like spots, adequate resin, the aroma is thick, numb the tongue, hard texture, sticky	√
CNA4	*A. sinensis*	10-year-old Chi-Nan germplasm by drilling for 12 months	Maoming, Guangdong province	Irregular strips, apparent brown resin spread throughout the surface, intense aroma, taste peppery, hard texture, sticky	√
CNA5	*A. sinensis*	3-year-old Chi-Nan germplasm by drilling for 12 months	Maoming, Guangdong province	Irregular pieces, black brown resin scatter like spots, saturated with resin, fragrance is elegant, spicy and numb, tough, sticky	x
CNA6	*A. sinensis*	20-year-old Chi-Nan germplasm by drilling for 18 months	Maoming, Guangdong province	Irregular pieces, black brown resin scatter like spots or stripe, quite strong aroma, little spicy and numb, tough, sticky	√
CNA7	*A. sinensis*	3-year-old Chi-Nan germplasm by drilling for 12 months	Maoming, Guangdong province	Irregular pieces, obvious black brown resin scatter like spots or stripe, cool feeling, taste peppery and numb, tough, soft and glutinous	√
OA1	*A. sinensis*	6-year-old trees induced by Agar-Wit^a^ for 18 months	Haikou, Hainan province	Irregular slices, brown resin bands alternate with yellow white wood stripe, pleasant fragrance, crisp	x
OA2	*A. sinensis*	6-year-old trees induced by Agar-Wit for 8 months	Danzhou, Hainan province	Irregular thin slices, saturated with resin brown resin, sweet fragrance, crisp	x
OA3	*A. sinensis*	Wild agarwood	Hainan province	Irregular pieces, massive protrusions and patches distribute throughout the appearance, slight aroma, soft	x
OA4	*A. sinensis*	Wild agarwood	Hainan province	Irregular pieces, many protrusions and patches distribute throughout the appearance, tawny resin scatter like spots, slight aroma, crisp	x
OA5	*A. sinensis*	5-year-old trees induced by BCD^b^ for 12 months	Maoming, Guangdong province	Irregular pieces or slices, tawny resin and white wood are distributed alternately, many fibers in the cross section, slight aroma, resilient	x
OA6	*A. sinensis*	5-year-old trees induced by BCD for about 12 months	Maoming, Guangdong province	Irregular pieces, brown resin scatter like spots, cheerful, aroma, crisp	x

^a^The abbreviation of whole-tree agarwood-inducing technique is Agar-Wit. ^b^The abbreviation of burning-chisel-drilling is BCD. “√”: the agarwood sample could sink in water. “x”: the agarwood sample could not sink in water.

**Table 2:** Thirteen distinguished compounds between CNA and OA in terms of 2-(2-phenylethyl)chromones.

Variable ID	Molecular formula	*p* value	Group	Proposed compound		
				Types	*A* ring	*B* ring
**6455**	C_19_H_18_O_4_	0.014	OA	FTPECs	1 OCH_3_	1 OCH_3_
**4531**	C_18_H_16_O_5_	0.024	OA	DEPECs		1 OCH_3_
**6289**	C_20_H_20_O_5_	0.028	OA	FTPECs	2 OCH_3_	1 OCH_3_
**5685**	C_19_H_18_O_5_	0.001	OA	FTPECs	1 OCH_3_	1 OH, 1 OCH_3_
**3225**	C_16_H_16_O_6_	0.005	OA	THPECs	2 OH	1 OH, 1 OCH_3_
**4729**	C_17_H_14_O_4_	0.034	OA	DEPECs	2 -O-	
**2045**	C_17_H_18_O_6_	0.005	OA	THPECs	Agarotetrol	
**6964**	C_18_H_16_O_3_	^*∗∗∗*^	CNA	FTPECs		1 OCH_3_
**7234**	C_17_H_14_O_2_	^*∗∗∗*^	CNA	FTPECs	2-(2-Phenylethyl)chromone	
**5255**	C_18_H_16_O_4_	^*∗∗∗*^	CNA	FTPECs		1 OH, 1 OCH_3_
**6936**	C_35_H_28_O_7_	^*∗∗∗*^	CNA	Dimeric-PECs		
**7180**	C_36_H_30_O_8_	^*∗∗∗*^	CNA	Dimeric-PECs		
**5072**	C_18_H_16_O_4_	^*∗∗∗*^	CNA	FTPECs		1 OH, 1 OCH_3_

^*∗∗∗*^indicates a significant difference between two groups (*p* < 0.001).

**Table 3:** Twelve distinguished compounds between CNA and OA in terms of violate constituents.

Peak	RI	Molecular formula	*p* value	Group	Proposed compound
570	1504	C_11_H_14_O_2_	^*∗∗∗*^	OA	4-(4-methoxyphenyl)-2-butanone
53	733	C_2_H_4_O_2_	0.002	OA	acetic acid
334	1248	C_10_H_12_O	0.022	OA	4-phenyl-2-butanone
64	750	C_3_H_6_O_2_	0.008	OA	1-hydroxy-2-propanone
180	966	C_7_H_6_O	0.047	OA	benzaldehyde
614	1526	C_10_H_8_O_2_	^*∗∗∗*^	CNA	2-methyl-4H-1-benzopyran-4-one
304	1207	C_8_H_8_O_2_	0.009	CNA	4-methoxybenzaldehyde
361	1290	C_8_H_10_O_2_	^*∗∗∗*^	CNA	4-methoxybenzenemethanol
223	1051	C_7_H_6_O_2_	^*∗∗∗*^	CNA	2-hydroxybenzaldehyde
286	1172	C_8_H_8_O_2_	0.001	CNA	1-(2-hydroxyphenyl)ethanone
1589	2618	C_17_H_14_O_2_	^*∗∗∗*^	CNA	2-(2-phenethyl)chromone
1557	2352	C_18_H_16_O_3_	^*∗∗∗*^	CNA	2-[2-(4′-methoxyphenyl)ethyl]chromone

^*∗∗∗*^indicates a significant difference between two groups (*p* < 0.001).

## Data Availability

All data included in this study are available upon request to the corresponding author.

## Abbreviations

CNA: Chi-Nan agarwood
OA: Ordinary agarwood
SHS-GC-MS: Static headspace sampling gas chromatography mass spectrometry
UPLC: Ultra-performance liquid chromatography
Q-Tof-MS: Quadrupole time-of-flight mass spectrometry
HRMS: High-resolution mass spectrometry
PCA: Principal components analysis
OPLS-DA: Orthogonal partial least squares discriminant analysis
BCD: Burning-chisel-drilling
Agar-Wit: Whole-tree agarwood-inducing technique
PC: Principal component
PECs: 2-(2-phenylethyl)chromones
THPECs: 5,6,7,8-tetrahydro-2-(2-phenylethyl)-chromones
DEPECs: 5,6,7,8-diepoxy-tetrahydro-2-(2-phenylethyl)chromones
EPECs: Epoxy-tetrahydro-2-(2-phenylethyl)chromones
FTPECs: Flindersia type 2-(2-phenethyl)chromones.

## Supplementary Materials

Supplementary Materials Tables S1–S2 and Figures S1–S3 in Supplementary Materials provide the details on comprehensive image analysis.
