# Supplementary material for: Remarkable Phytochemical Characteristics of Chi-Nan Agarwood Induced from New-Found Chi-Nan Germplasm of Aquilaria sinensis Compared with Ordinary Agarwood
Source: Int J Anal Chem. 2021 Apr 10;2021:5593730. doi: 10.1155/2021/5593730 (PMC8053051; doi:10.1155/2021/5593730)
Supplement: Supplementary Materials — Tables S1–S2 and Figures S1–S3 in Supplementary Materials provide the details on comprehensive image analysis. [file 5593730.f1.docx]

**Table S1 Identified 2-(2-phenethyl)chromones detected by LC-Q/Tof-MS**

| NO. | RT (min) | [M+H]^+^ | Molecular formula | Error ppm | Fragment ions | A-ring | B-ring | Proposed compound |
| --- | --- | --- | --- | --- | --- | --- | --- | --- |
| 1 | 3.34 | 365.1232 | C_18_H_20_O_8_ | -1.1 | 347.1135; 329.1034; 137.0602 | 4OH | OH; OCH_3_ | 2-[2-(3-hydroxy-4-methoxyphenyl)ethyl]-5,6,7,8-tetrahydroxy-5,6,7,8-tetrahydrochromone (isomer1) |
| 2 | 3.78 | 365.1232 | C_18_H_20_O_8_ | -1.1 | 347.1124; 329.1032; 137.0600 | 4OH | OH; OCH_3_ | 2-[2-(3-hydroxy-4-methoxyphenyl)ethyl]-5,6,7,8-tetrahydroxy-5,6,7,8-tetrahydrochromone (isomer2) |
| 3 | 5.42 | 319.1183 | C_17_H_18_O_6_ | 0.3 | 301.1080; 283.0957; 255.1019; 227.1081;121.0651 | 4OH |  | Agarotetrol |
| 4 | 6.12 | 349.1290 | C_18_H_20_O_7_ | 0.8 | 331.1184;313.1078; 121.0650; | 4OH | OCH_3_ | 2-[2-(4'-methoxyphenyl)ethyl]-5,6,7,8-tetrahydroxy-5,6,7,8-tetrahydrochromone (new compound) |
| 5 | 6.35 | 319.1183 | C_17_H_18_O_6_ | 0.3 | 301.1085; 283.0968; 225.1016; 164.0470 | 4OH |  | Agarotetrol (isomer1) |
| 6 | 6.79 | 347.1134 | C_18_H_18_O_7_ | 0.9 | 329.1028; 311.1841; 137.0619 | -O-; 2OH | OH; OCH_3_ | 2-[2-(3'-hydroxy-4'-methoxyphenyl)ethyl]-7,8-epoxy-5,6-dihydroxy-5,6,7,8-tetrahydrochromone(isomer1) |
| 7 | 8.64 | 329.1023 | C_16_H_16_O_6_ | -0.6 | 301.1063; 137.0598; | 2-O- | OH; OCH_3_ | 5,6:7,8-diepoxy-2-[2-(3-hydroxy-4-methoxyphenyl)ethyl]-5,6,7,8-tetrahydrochromone(isomer1) |
| 8 | 9.26 | 331.1183 | C_18_H_18_O_6_ | 0.3 | 313.1079; 121.0652 | -O-; 2OH | OCH_3_ | 2-[2-(4'-methoxyphenyl)ethyl]- 7,8-epoxy-5,6-dihydroxy-5,6,7,8-tetrahydrochromone(isomer2) |
| 9 | 10.98 | 313.1075 | C_18_H_16_O_5_ | -0.3 | 121.0657 | 2-O- | OCH_3_ | 5,6:7,8-diepoxy-2-[2-(4-methoxyphenyl)ethyl]-5,6,7,8-tetrahydrochromone(isomer1) |
| 10 | 11.29 | 337.0844 | C_17_H_17_ClO_5_ | -0.3 | 319.0742; 301.0639; 283.0982 | 3OH; Cl |  | 8-chloro-5,6,7-trihydroxy-5,6,7,8-tetrahydro-2-(2-phenylethyl)chromone(isomer1) |
| 11 | 11.86 | 343.1180 | C_19_H_18_O_6_ | -0.6 | 205.5490; 137.0598 | OH, OCH_3_ | OH, OCH_3_ | 6-hydroxy-7-methoxy-2-[2-(3'-hydroxy-4'-methoxyphenyl)ethyl]chromone |
| 12 | 11.81 | 331.1183 | C_18_H_18_O_6_ | 0.3 | 313.1087; 285.1126; 121.0648 | -O-; 2OH | OCH_3_ | 2-[2-(4'-methoxyphenyl)ethyl]- 7,8-epoxy-5,6-dihydroxy-5,6,7,8-tetrahydrochromone(isomer3) |
| 13 | 12.11 | 301.1078 | C_17_H_16_O_5_ | 0.7 | 283.0971; 255.1020 | -O-; 2OH |  | 2-(2-phenylethyl)-7,8-epoxy-5,6-dihydroxy-5,6,7,8-tetrahydrochromone |
| 14 | 12.47 | 313.1079 | C_18_H_16_O_5_ | 1.0 | 285.1130; 121.0651; | 2-O- | OCH_3_ | 5,6:7,8-diepoxy-2-[2-(4-methoxyphenyl)ethyl]-5,6,7,8-tetrahydrochromone(isomer2) |
| 15 | 13.04 | 313.1076 | C_18_H_16_O_5_ | 0 | 137.0606; | OH | OH, OCH_3_ | 6-hydroxy-2-[2-(3'-hydroxy-4'-methoxyphenyl)ethyl]chromone(isomer1) |
| 16 | 14.38 | 313.1077 | C_18_H_16_O_5_ | 0.3 | 285.1127; 121.0650; | 2-O- | OCH_3_ | 5,6:7,8-diepoxy-2-[2-(4-methoxyphenyl)ethyl]-5,6,7,8-tetrahydrochromone(Oxidoagarochromone B) |
| 17 | 14.95 | 283.0970 | C_17_H_14_O_4_ | 0 | 255.1019;227.1072 | 2-O- |  | 5,6:7,8-diepoxy-2-[2-(4-methoxyphenyl)ethyl]-5,6,7,8-tetrahydrochromone(Oxidoagarochromone A) |
| 18 | 17.54 | 327.1235 | C_19_H_18_O_5_ | 0.9 | 121.0660 | OH, OCH_3_ | OCH_3_ | 6-hydroxy-7-methoxy-2-[2-(4'-methoxyphenyl)ethyl]chromone(isomer1) |
| 19 | 17.77 | 327.1232 | C_19_H_18_O_5_ | 0 | 191.0704; 137.0601 | OCH_3_ | OH, OCH_3_ | 6-methoxy-2-[2-(3'-hydroxy-4'-methoxyphenyl)ethyl]chromone(isomer1) |
| 22 | 19.14 | 297.1128 | C_18_H_16_O_4_ | 0.3 | 121.0652 | OH | OCH_3_ | 6-hydroxy-2-[2-(4'-methoxyphenyl)ethyl]chromone(isomer1) |
| 21 | 19.84 | 267.1024 | C_17_H_14_O_3_ | 1.1 | 176.0476; 137.0238 | OH |  | 6-hydroxy-2-(2-phenylethyl)chromone(isomer1) |
| 22 | 21.07 | 341.1390 | C_20_H_20_O_5_ | 0.3 | 121.0650 | 2OCH_3_ | OCH_3_ | 6,7-dimethoxy-2-[2-(4'-methoxyphenyl)ethyl]chromone(isomer1) |
| 23 | 23.64 | 281.1182 | C_18_H_16_O_3_ | 0.3 | 121. 0652 |  | OCH_3_ | 2-[2-(4'-methoxyphenyl)ethyl]chromone |
| 24 | 24.21 | 251.1079 | C_17_H_14_O_2_ | 3 | 121.0282 |  |  | 2-(2-phenylethyl)chromone |
| 25 | 24.57 | 311.1284 | C_19_H_18_O_4_ | 0.3 | 121.0646 | OCH_3_ | OCH_3_ | 6-methoxy-2-[2-(4'-methoxyphenyl)ethyl]chromone(isomer1) |
| 26 | 24.96 | 281.1182 | C_18_H_16_O_3_ | 0.3 | 190.0630; 151.0394 | OCH_3_ |  | 6-methoxy-2-(2-phenylethyl)chromone(isomer1) |
| 27 | 16.44 | 297.1127 | C_18_H_16_O_4_ | 0 | 161.0596; 137.0599 |  | OH; OCH_3_ | 2-[2-(2'-hydroxy-4'-methoxyphenyl)ethyl]chromone |
| 28 | 17.06 | 297.1129 | C_18_H_16_O_4_ | 0.7 | 160.0518; 137.0599 |  | OH; OCH_3_ | 2-[2-(3'-hydroxy-4'-methoxyphenyl)ethyl]chromone |
| 29 | 18.6 | 267.1021 | C_17_H_14_O_3_ | 0 | 161.0611; 173.5422; 107.0485 |  | OH | 2-[2-(4'-hydroxyphenyl)ethyl]chromone(isomer1) |
| 30 | 16.13 | 267.1024 | C_17_H_14_O_3_ | 1.1 | 161.0600;107.0493 |  | OH | 2-[2-(4'-hydroxyphenyl)ethyl]chromone(isomer2) |

**Table S2** **Identified compounds detected by SHS-GC-MS**

| **No.** | **RI** | **Compounds** | **Relative percentage content / %** | | | | | | | | | | | | | |
| --- | --- | --- | --- | --- | --- | --- | --- | --- | --- | --- | --- | --- | --- | --- | --- | --- |
|  |  |  | **CNA1** | **CNA2** | **CNA3** | **CNA8** | **CNA4** | **CNA5** | **CNA6** | **CNA7** | **OA6** | **OA5** | **OA1** | **OA2** | **OA3** | **OA4** |
| 1 | 666 | 2,3-Butanedione |  |  |  |  |  | 0.147 |  |  |  |  |  |  | 0.685 |  |
| 2 | 836 | 3-Furaldehyde | 0.469 | 0.432 | 0.344 | 0.646 | 0.274 | 0.333 | 0.511 | 0.397 | 0.993 | 4.365 | 0.539 | 0.374 | 3.871 | 3.368 |
| 3 | 887 | 3-Heptanone |  |  |  |  |  |  |  |  | 0.328 | 0.881 | 0.114 |  | 0.616 | 0.489 |
| 4 | 895 | Styrene | 0.160 | 0.211 | 0.464 | 0.209 | 0.303 | 0.152 | 0.552 | 0.555 | 0.507 | 0.458 | 0.332 | 0.335 | 0.392 | 0.224 |
| 5 | 902 | Heptanal |  | 0.038 |  |  |  |  |  |  |  | 0.254 |  | 0.035 | 0.154 | 0.117 |
| 6 | 966 | Benzaldehyde |  | 4.017 |  | 2.962 |  |  |  |  | 5.855 | 9.003 | 2.935 |  | 6.036 | 5.260 |
| 7 | 984 | Phenol | 0.120 | 0.191 | 0.127 |  |  | 0.246 |  |  |  |  |  |  |  |  |
| 8 | 1025 | Benzene, 1-methoxy-4-methyl- | 0.068 | 0.068 | 0.120 | 0.095 | 0.084 | 0.093 | 0.321 | 0.303 | 0.135 | 0.182 | 0.091 |  | 0.222 | 0.183 |
| 9 | 1029 | 1-Hexanol, 2-ethyl- | 0.162 |  | 0.181 | 0.196 | 0.185 | 0.124 | 0.203 | 0.180 |  |  | 0.197 | 0.108 |  |  |
| 10 | 1041 | Benzyl alcohol | 1.280 | 1.411 | 2.394 | 0.650 | 2.180 | 1.535 | 2.594 | 4.408 | 1.963 | 1.459 | 0.737 | 0.766 | 0.958 | 0.533 |
| 11 | 1051 | Benzaldehyde, 2-hydroxy- | 0.756 | 0.985 | 1.243 |  | 1.108 | 1.021 | 2.170 | 1.883 |  |  |  |  | 0.278 |  |
| 12 | 1073 | Acetophenone | 0.404 | 0.628 | 0.607 | 0.668 | 0.541 | 0.357 | 0.902 | 0.949 | 1.585 | 1.589 | 0.840 | 0.886 | 1.319 | 0.787 |
| 13 | 1095 | Phenol, 2-methoxy- |  | 0.092 |  |  |  |  | 0.242 | 0.250 |  |  |  |  |  |  |
| 14 | 1105 | Nonanal | 0.074 | 0.150 | 0.067 | 0.077 | 0.100 |  | 0.168 | 0.128 | 0.124 | 0.346 | 0.097 | 0.105 | 0.220 | 0.171 |
| 15 | 1119 | Benzene, 1-ethyl-4-methoxy- |  |  |  |  |  |  | 0.057 | 0.059 | 0.237 | 0.159 | 0.148 |  | 0.318 | 0.279 |
| 16 | 1159 | Benzene, 1-ethenyl-4-methoxy- | 0.284 | 0.344 | 0.583 | 0.809 | 0.255 | 0.251 | 0.210 | 0.259 | 1.124 | 0.779 | 0.737 | 0.312 | 1.200 | 0.842 |
| 17 | 1169 | Benzenepropanal |  |  |  |  |  |  |  |  | 0.186 |  | 0.146 | 0.439 |  |  |
| 18 | 1172 | Ethanone, 1-(2-hydroxyphenyl)- | 0.826 | 2.129 | 0.966 | 1.265 | 1.068 | 3.055 | 1.057 | 0.926 | 0.292 |  | 0.170 |  | 0.296 |  |
| 19 | 1207 | Benzaldehyde, 3-methoxy- | 0.288 | 0.152 | 0.466 | 4.032 | 0.447 | 3.864 | 1.304 | 1.226 | 4.889 | 5.274 | 0.119 | 1.562 | 6.747 | 5.084 |
| 20 | 1236 | 6,7-Dimethyl-1,2,3,5,8,8a-hexahydronaphthalene | 0.558 |  | 1.647 | 1.073 | 0.866 | 3.93 |  |  | 1.822 | 1.774 | 1.462 | 6.54 | 1.386 | 1.524 |
| 21 | 1248 | 2-Butanone, 4-phenyl- | 2.343 | 5.091 | 2.869 | 1.975 | 1.696 | 3.193 | 4.772 | 2.692 | 8.616 | 8.810 | 5.052 | 9.054 | 5.454 | 3.766 |
| 22 | 1282 | Benzene, 4-(2-butenyl)-1,2-dimethyl-, |  |  | 0.342 | 0.246 | 0.237 | 0.167 | 0.183 | 0.225 | 0.366 | 0.503 | 0.423 | 0.604 | 0.533 | 0.632 |
| 23 | 1290 | Benzenemethanol, 4-methoxy- | 0.839 | 0.545 | 1.476 | 0.228 | 0.957 | 1.303 | 1.694 | 2.192 | 0.375 |  | 0.161 |  |  |  |
| 24 | 1313 | 3-Penten-2-one, 4-phenyl- |  |  |  |  |  |  |  |  | 0.599 | 0.493 | 0.073 | 0.044 | 0.133 | 0.071 |
| 25 | 1323 | Naphthalene, 2-methyl- |  |  |  | 0.037 |  |  |  |  | 0.051 | 0.105 | 0.020 | 0.028 | 0.106 | 0.103 |
| 26 | 1328 | 1H-Indene, 1,1,3-trimethyl- |  |  | 0.026 | 0.037 | 0.021 |  | 0.038 | 0.038 | 0.027 | 0.075 | 0.032 | 0.098 | 0.073 | 0.081 |
| 27 | 1349 | 4-(2-Methyl-cyclohex-1-enyl)-but-3-en-2-one |  | 0.103 | 0.104 |  | 0.141 |  | 0.180 |  |  |  |  |  |  |  |
| 28 | 1358 | 4-Acetylanisole |  | 0.237 | 0.427 | 0.884 |  |  | 0.703 | 0.606 | 0.911 | 0.700 | 0.592 | 0.218 | 1.242 | 0.976 |
| 29 | 1362 | 3-Buten-2-one, 4-phenyl- |  | 0.135 |  |  |  |  |  |  | 0.036 |  | 0.242 | 0.220 |  |  |
| 30 | 1396 | 1H-2-Benzopyran-1-one, 3,4-dihydro- | 0.065 | 0.117 | 0.121 |  | 0.078 | 0.066 | 0.217 | 0.167 | 0.175 |  | 0.221 | 0.245 |  |  |
| 31 | 1563 | 2(3H)-Naphthalenone, 4,4a,5,6,7,8-hexahydro-4,4a-dimethyl-6-(1-methylethylidene)-, (4R-cis)- |  |  |  |  | 0.733 |  |  |  |  |  | 2.001 | 1.750 |  | 2.144 |
| 32 | 1402 | Cyclohexane, 1-ethenyl-1-methyl-2,4-bis(1-methylethenyl)-, [1S-(1.alpha.,2.beta.,4.beta.)]- | 0.156 | 0.826 |  | 0.113 | 0.918 |  |  |  | 0.091 |  | 0.132 |  |  |  |
| 33 | 1432 | 1H-3a,7-Methanoazulene, 2,3,4,7,8,8a-hexahydro-3,6,8,8-tetramethyl-, [3R-(3.alpha.,3a.beta.,7.beta.,8a.alpha.)]- | 0.233 |  |  |  |  |  |  |  |  |  |  |  |  |  |
| 34 | 1452 | 1,1,7,7a-Tetramethyl-1a,2,6,7,7a,7b-hexahydro-1H-cyclopropa[a]naphthalene | 0.153 |  |  |  | 0.465 |  | 0.256 | 0.250 |  |  |  |  |  |  |
| 35 | 1457 | Vanillin | 0.833 | 0.551 | 0.457 | 0.583 | 1.373 | 0.346 | 2.409 | 2.669 | 0.333 | 1.171 | 0.251 | 0.144 | 2.619 | 2.199 |
| 36 | 1477 | (4S,4aR,6R)-4,4a-Dimethyl-6-(prop-1-en-2-yl)-1,2,3,4,4a,5,6,7-octahydronaphthalene | 0.695 | 0.328 | 0.498 | 0.845 | 0.371 |  |  | 0.598 | 0.199 |  | 0.348 |  | 0.232 |  |
| 37 | 1482 | 4a,8-Dimethyl-2-(prop-1-en-2-yl)-1,2,3,4,4a,5,6,7-octahydronaphthalene | 0.815 | 0.699 |  |  |  |  |  |  |  |  |  | 0.698 |  | 1.495 |
| 38 | 1486 | Benzene, 1-(1,5-dimethyl-4-hexenyl)-4-methyl- | 0.398 | 0.410 |  |  |  |  |  |  |  |  |  |  |  |  |
| 39 | 1505 | Naphthalene, decahydro-4a-methyl-1-methylene-7-(1-methylethenyl)-, [4aR-(4a.alpha.,7.alpha.,8a.beta.)]- | 1.136 |  |  |  |  |  | 1.159 | 0.777 |  |  |  |  |  |  |
| 40 | 1485 | 2H-2a,7-Methanoazuleno[5,6-b]oxirene, octahydro-3,6,6,7a-tetramethyl- |  |  | 3.908 | 3.385 | 3.131 |  |  | 3.123 | 4.670 | 4.096 |  | 8.162 | 6.156 | 6.541 |
| 41 | 1498 | Azulene, 1,2,3,3a,4,5,6,7-octahydro-1,4-dimethyl-7-(1-methylethenyl)-, [1R-(1.alpha.,3a.beta.,4.alpha.,7.beta.)]- |  | 0.885 | 0.580 | 1.041 | 1.064 |  |  |  | 0.877 |  | 1.694 |  |  |  |
| 42 | 1503 | 3-Isopropyl-6,8a-dimethyl-1,2,4,5,8,8a-hexahydroazulene |  |  | 0.419 |  |  |  |  | 0.793 |  |  |  |  |  |  |
| 43 | 1505 | Naphthalene, decahydro-4a-methyl-1-methylene-7-(1-methylethylidene)-, (4aR-trans)- | 0.816 | 1.237 | 0.498 | 0.646 | 0.897 | 0.371 | 1.865 | 0.641 | 1.046 |  |  |  | 1.037 | 1.238 |
| 44 | 1510 | (3R,5aS,9aR)-2,2,5a,9-Tetramethyl-3,4,5,5a,6,7-hexahydro-2H-3,9a-methanobenzo[b]oxepine |  | 0.581 |  |  |  |  |  |  |  |  |  |  |  |  |
| 45 | 1520 | (2R,8R,8aS)-8,8a-Dimethyl-2-(prop-1-en-2-yl)-1,2,3,7,8,8a-hexahydronaphthalene | 0.163 | 0.120 | 0.153 |  | 0.312 |  |  |  |  | 0.273 | 0.214 |  | 0.239 | 0.230 |
| 46 | 1524 | Azulene, 1,2,3,5,6,7,8,8a-octahydro-1,4-dimethyl-7-(1-methylethenyl)-, [1S-(1.alpha.,7.alpha.,8a.beta.)]- |  |  |  |  |  |  | 0.900 |  |  |  |  |  |  |  |
| 47 | 1526 | 4H-1-Benzopyran-4-one, 2-methyl- | 0.848 | 0.862 | 1.013 |  | 1.960 | 0.712 | 2.411 | 1.705 |  |  |  |  |  |  |
| 48 | 1532 | Kessane | 3.818 |  |  |  |  | 1.062 |  |  |  |  |  |  |  |  |
| 49 | 1536 | 2H-3,9a-Methano-1-benzoxepin, octahydro-2,2,5a,9-tetramethyl-, [3R-(3.alpha.,5a.alpha.,9.alpha.,9a.alpha.)]- | 1.356 |  | 0.398 |  | 0.591 | 0.282 |  |  | 0.710 | 0.701 | 0.851 | 0.948 | 0.809 | 0.884 |
| 50 | 1652 | 2-Naphthalenemethanol, decahydro-.alpha.,.alpha.,4a-trimethyl-8-methylene-, [2R-(2.alpha.,4a.alpha.,8a.beta.)]- |  | 5.150 |  |  |  |  |  |  |  |  |  |  |  |  |
| 51 | 1501 | (5R,10R)-10-Methyl-6-methylene-2-(propan-2-ylidene)spiro[4.5]dec-7-ene |  |  |  |  |  |  |  |  |  |  |  | 0.343 | 0.488 | 0.596 |
| 52 | 1504 | 2-Butanone, 4-(4-methoxyphenyl)- |  |  |  | 1.933 |  | 0.604 |  |  | 6.980 | 7.093 | 4.158 | 1.679 | 6.613 | 6.113 |
| 53 | 1514 | 4a,5-Dimethyl-3-(prop-1-en-2-yl)-1,2,3,4,4a,5,6,7-octahydronaphthalen-1-ol |  |  |  | 0.612 |  |  |  |  | 0.320 | 0.615 | 0.425 | 0.371 |  |  |
| 53 | 1545 | .alpha.-Guaiene |  |  |  |  |  |  |  |  |  |  | 1.694 | 0.215 | 0.180 |  |
| 54 | 1549 | 3,5,11-Eudesmatriene | 2.246 | 2.714 | 2.154 | 1.521 | 2.459 | 1.778 | 0.735 |  | 0.670 |  | 1.680 | 1.948 |  | 0.660 |
| 55 | 1583 | .alpha.-Santalol | 4.999 |  |  | 0.932 | 11.182 | 3.634 | 2.362 | 2.978 | 1.059 |  | 2.845 | 1.330 |  |  |
| 56 | 1574 | (4aS,8R)-4a,8-Dimethyl-4,4a,5,6,7,8-hexahydronaphthalen-2(3H)-one |  |  | 0.354 |  |  |  |  | 0.223 |  |  |  |  |  |  |
| 57 | 1544 | Selina-3,7(11)-diene |  |  |  |  |  |  |  |  |  | 0.385 |  |  | 0.613 | 0.658 |
| 58 | 1591 | (8R,8aS)-8,8a-Dimethyl-2-(propan-2-ylidene)-1,2,3,7,8,8a-hexahydronaphthalene | 0.557 | 0.316 | 0.465 | 0.876 | 0.622 | 0.831 |  |  | 0.376 | 0.331 | 1.770 | 1.106 | 1.021 | 0.475 |
| 59 | 1610 | 3H-3a,7-Methanoazulene, 2,4,5,6,7,8-hexahydro-1,4,9,9-tetramethyl-, [3aR-(3a.alpha.,4.beta.,7.alpha.)]- | 0.484 |  |  |  |  | 1.472 |  |  |  |  |  |  |  |  |
| 60 | 1686 | 2-((2R,4aR,8aR)-4a,8-Dimethyl-1,2,3,4,4a,5,6,8a-octahydronaphthalen-2-yl)acrylaldehyde |  | 0.577 |  |  |  |  |  |  |  |  |  |  |  |  |
| 61 | 1693 | 6-Isopropenyl-4,8a-dimethyl-1,2,3,5,6,7,8,8a-octahydro-naphthalen-2-ol | 0.764 | 0.747 |  |  |  |  |  |  |  |  |  |  |  |  |
| 62 | 1577 | 1,7-Dimethyl-4-(propan-2-ylidene)tricyclo[4.4.0.02,7]decan-3-one |  | 1.268 |  |  |  |  |  |  |  |  |  |  |  |  |
| 63 | 1671 | Tricyclo[4.4.0.0(2,7)]dec-8-ene-3-methanol, .alpha.,.alpha.,6,8-tetramethyl-, stereoisomer |  |  | 1.908 |  |  |  |  |  |  |  |  |  |  |  |
| 64 | 1686 | (E)-3-((4S,7R,7aR)-3,7-Dimethyl-2,4,5,6,7,7a-hexahydro-1H-inden-4-yl)-2-methylacrylaldehyde |  |  | 0.283 |  | 0.512 |  | 0.347 |  |  |  | 0.497 |  |  |  |
| 65 | 1673 | 2-Butanone, 4-(4-hydroxy-3-methoxyphenyl)- |  |  |  |  |  |  |  |  |  |  |  |  | 1.405 | 1.422 |
| 66 | 1751 | 2-((2R,4aR,8aS)-4a-Methyl-8-methylenedecahydronaphthalen-2-yl)prop-2-en-1-ol | 0.278 | 9.067 | 0.801 | 0.538 | 1.868 |  |  | 0.642 | 1.670 |  | 0.737 | 0.764 | 0.670 | 0.925 |
| 67 | 1493 | Naphthalene, 1,2,4a,5,8,8a-hexahydro-4,7-dimethyl-1-(1-methylethyl)-, (1.alpha.,4a.beta.,8a.alpha.) |  |  |  |  |  |  | 0.511 |  |  | 1.029 |  |  | 1.730 |  |
| 68 | 1632 | 2-Naphthalenemethanol, 1,2,3,4,4a,5,6,7-octahydro-.alpha.,.alpha.,4a,8-tetramethyl-, (2R-cis)- | 2.114 | 1.562 |  |  |  | 0.788 | 0.502 |  | 0.541 | 0.337 | 0.458 | 0.425 | 0.293 | 0.296 |
| 69 | 1636 | Agarospirol | 2.053 | 1.822 | 1.637 | 1.892 | 0.191 | 1.914 | 5.978 | 2.031 | 0.979 | 0.112 | 1.980 | 1.571 | 0.515 | 0.181 |
| 70 | 1645 | (-)-Aristolene |  |  |  |  | 0.943 |  |  |  |  |  |  | 2.422 |  |  |
| 71 | 1652 | 2-((2R,8R,8aS)-8,8a-Dimethyl-1,2,3,4,6,7,8,8a-octahydronaphthalen-2-yl)propan-2-ol |  |  |  |  |  | 2.293 |  |  |  |  | 3.704 | 4.095 | 0.515 |  |
| 72 | 1671 | 1H-Cycloprop[e]azulen-7-ol, decahydro-1,1,7-trimethyl-4-methylene-, [1ar-(1a.alpha.,4a.alpha.,7.beta.,7a.beta.,7b.alpha.)]- | 1.472 |  | 0.288 |  | 1.136 | 0.412 | 0.390 | 0.441 |  |  |  |  |  |  |
| 73 | 1661 | 1H-Cyclopropa[a]naphthalene, 1a,2,3,3a,4,5,6,7b-octahydro-1,1,3a,7-tetramethyl-, [1aR-(1a.alpha.,3a.alpha.,7b.alpha.)]- |  |  | 0.844 | 1.271 |  |  | 0.363 | 1.022 | 2.363 | 1.937 | 2.179 | 4.225 | 1.034 | 1.078 |
| 74 | 1623 | 2-((2S,4aR)-4a,8-Dimethyl-1,2,3,4,4a,5,6,7-octahydronaphthalen-2-yl)propan-2-ol |  |  |  |  | 0.933 | 1.011 |  |  |  |  |  |  |  |  |
| 75 | 1639 | Hinesol |  |  |  |  |  |  |  |  | 0.856 | 0.224 | 0.552 | 0.690 | 0.450 | 0.271 |
| 76 | 1676 | 5-Azulenemethanol, 1,2,3,4,5,6,7,8-octahydro-.alpha.,.alpha.,3,8-tetramethyl-, acetate, [3S-(3.alpha.,5.alpha.,8.alpha.)]- | 0.688 |  | 1.628 | 4.181 | 0.786 | 2.763 |  |  | 3.420 | 0.796 | 2.998 |  | 0.874 | 1.754 |
| 77 | 1595 | Aristol-1(10)-en-9-ol |  | 0.370 |  |  |  |  |  |  |  |  |  |  |  |  |
| 78 | 1616 | Selina-6-en-4-ol |  | 0.206 |  |  |  |  |  |  |  |  |  |  |  |  |
| 79 | 1619 | 7-epi-a-Eudesmol |  | 0.363 |  |  |  |  |  |  |  |  |  |  |  |  |
| 80 | 1684 | Guaiol | 2.341 | 0.567 |  |  |  |  |  |  |  |  |  |  |  |  |
| 81 | 1548 | Naphthalene, 1,6-dimethyl-4-(1-methylethyl)- |  |  |  | 0.059 |  |  |  |  |  |  |  |  |  | 0.124 |
| 82 | 1697 | 1(2H)-Naphthalenone, 3,4,4a,5,6,7-hexahydro-4a,5-dimethyl-3-(1-methylethenyl)-, [3S-(3.alpha.,4a.alpha.,5.alpha.)]- | 0.647 | 0.754 |  | 0.557 | 0.820 | 0.447 |  |  | 0.653 |  |  |  |  |  |
| 83 | 1693 | 7R,8R-8-Hydroxy-4-isopropylidene-7-methylbicyclo[5.3.1]undec-1-ene | 1.382 |  |  |  |  |  | 0.874 |  |  |  |  |  |  |  |
| 84 | 1722 | 2-(4a,8-Dimethyl-1,2,3,4,4a,5,6,7-octahydro-naphthalen-2-yl)-prop-2-en-1-ol |  | 2.322 |  |  | 2.712 | 1.408 |  |  | 1.216 |  | 0.844 |  |  |  |
| 85 | 1824 | 2aS,3aR,5aS,9bR)-2a,5a,9-Trimethyl-2a,4,5,5a,6,7,8,9b-octahydro-2H-naphtho[1,2-b]oxireno[2,3-c]furan |  |  |  | 3.000 |  | 0.828 |  | 0.353 | 0.691 |  | 1.032 | 1.311 | 0.461 | 0.760 |
| 86 | 1891 | Longifolenaldehyde | 1.129 | 0.295 | 0.373 | 0.474 | 1.546 | 0.380 | 0.769 | 0.232 |  |  | 1.018 | 0.810 | 0.421 | 0.501 |
| 87 | 1722 | Ledene alcohol |  |  | 0.949 |  |  |  |  | 0.863 |  |  |  | 0.629 |  |  |
| 88 | 1758 | Cadina-1(10),6,8-triene |  |  |  | 0.253 |  |  |  |  |  |  |  |  |  | 0.090 |
| 89 | 1797 | 2(3H)-Naphthalenone, 4,4a,5,6,7,8-hexahydro-4a,5-dimethyl-3-(1-methylethylidene)-, (4ar-cis)- | 2.388 | 1.021 | 4.885 | 0.840 | 1.666 | 2.274 | 0.955 | 1.214 |  |  | 1.133 | 0.994 |  |  |
| 90 | 1713 | 1-Naphthalenol, 5,6,7,8-tetrahydro-2,5-dimethyl-8-(1-methylethyl)- |  |  |  | 1.812 |  |  |  |  |  |  |  | 0.198 |  |  |
| 91 | 1743 | Ylangenal |  |  |  |  |  |  |  |  |  |  | 0.627 |  |  |  |
| 92 | 1760 | 24-Noroleana-3,12-diene |  | 0.462 |  | 0.876 | 0.444 |  |  |  |  |  | 0.453 |  |  |  |
| 93 | 1818 | 7-(2-Hydroxypropan-2-yl)-1,4a-dimethyldecahydronaphthalen-1-ol | 0.139 | 2.122 |  |  |  |  |  |  |  |  |  |  |  |  |
| 94 | 1810 | Cryptomeridiol |  | 0.753 |  |  |  |  |  |  | 0.373 |  |  |  |  |  |
| 95 | 1824 | Cyclobutane, tetrakis(1-methylethylidene)- | 0.635 | 0.255 | 0.749 |  | 0.803 |  | 0.392 |  |  |  |  |  |  |  |
| 96 | 1977 | (4aR,5S)-1-Hydroxy-4a,5-dimethyl-3-(propan-2-ylidene)-4,4a,5,6,7,8-hexahydronaphthalen-2(3H)-one | 0.270 |  |  |  | 0.248 |  |  |  |  |  |  |  |  |  |
| 97 | 1921 | 2(1H)-Naphthalenone, 4a,5,6,7,8,8a-hexahydro-6-[1-(hydroxymethyl)ethenyl]-4,8a-dimethyl-, [4ar-(4a.alpha.,6.alpha.,8a.beta.)]- |  |  | 0.293 |  | 0.139 |  |  |  |  |  |  |  |  |  |
| 98 | 1955 | 5,8-Dihydroxy-4a-methyl-4,4a,4b,5,6,7,8,8a,9,10-decahydro-2(3H)-phenanthrenone | 0.112 |  | 0.505 |  | 0.160 |  |  | 0.106 |  |  | 0.311 |  |  |  |
| 99 | 1978 | (4aR,5S)-1-Hydroxy-4a,5-dimethyl-3-(propan-2-ylidene)-4,4a,5,6-tetrahydronaphthalen-2(3H)-one | 0.238 | 0.182 | 0.553 |  | 0.225 | 0.186 |  |  | 0.255 |  | 0.928 |  |  |  |
| 100 | 2348 | 2-Phenethyl-4H-chromen-4-one | 3.509 | 5.777 | 6.483 | 1.949 | 4.127 | 6.583 | 5.776 | 5.094 |  |  | 0.135 |  |  |  |
| 101 | 2617 | 2-(4-Methoxyphenethyl)-4H-chromen-4-one | 1.986 | 2.153 | 3.094 |  | 1.465 | 2.921 | 2.524 | 2.668 |  |  |  |  |  |  |

**Figure S1** The pictures of 13 batches samples.


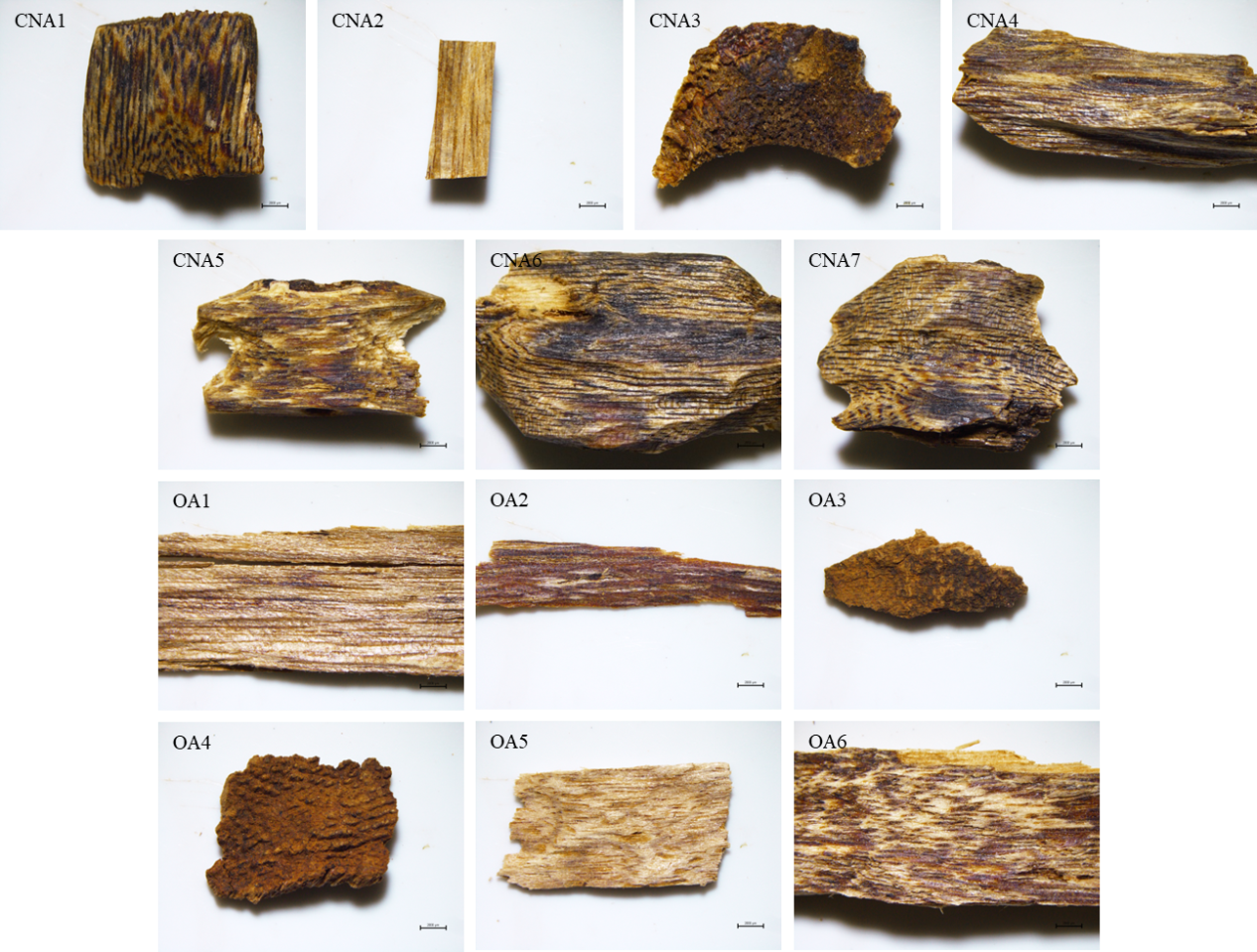


In order to prove our speculation, we also analyzed a wild Chi-Nan agarwood sample (Chi-Nan8 provided by collector) in the same conditions and the corresponding results shown in Figure S2-3.

**Figure S2** The total ion chromatograms of representative agarwood samples (OA1, CNA1 and CNA8) analyzed by LC-Q/Tof-MS.

**Figure S3** The total ion chromatograms of representative agarwood samples (OA1, CNA1 and CNA8) analyzed by GC-MS.
